# Soil surface temperatures reveal moderation of the urban heat island effect by trees and shrubs

**DOI:** 10.1038/srep33708

**Published:** 2016-09-19

**Authors:** J. L. Edmondson, I. Stott, Z. G. Davies, K. J. Gaston, J. R. Leake

**Affiliations:** 1 Department of Animal and Plant Sciences, University of Sheffield, Sheffield S10 2TN, U.K; 2 Max-Planck Odense Center, Department of Biology, University of Southern Denmark, Campusvej 55, 5230 Odense M, Denmark; 3 Durrell Institute of Conservation and Ecology (DICE), School of Anthropology and Conservation, University of Kent, Canterbury, Kent CT2 7NR, UK; 4 Environment and Sustainability Institute, University of Exeter, Penryn, Cornwall TR10 9FE, U.K

## Abstract

Urban areas are major contributors to air pollution and climate change, causing impacts on human health that are amplified by the microclimatological effects of buildings and grey infrastructure through the urban heat island (UHI) effect. Urban greenspaces may be important in reducing surface temperature extremes, but their effects have not been investigated at a city-wide scale. Across a mid-sized UK city we buried temperature loggers at the surface of greenspace soils at 100 sites, stratified by proximity to city centre, vegetation cover and land-use. Mean daily soil surface temperature over 11 months increased by 0.6 °C over the 5 km from the city outskirts to the centre. Trees and shrubs in non-domestic greenspace reduced mean maximum daily soil surface temperatures in the summer by 5.7 °C compared to herbaceous vegetation, but tended to maintain slightly higher temperatures in winter. Trees in domestic gardens, which tend to be smaller, were less effective at reducing summer soil surface temperatures. Our findings reveal that the UHI effects soil temperatures at a city-wide scale, and that in their moderating urban soil surface temperature extremes, trees and shrubs may help to reduce the adverse impacts of urbanization on microclimate, soil processes and human health.

The rapid global expansion in the areal extent of urban land is set to continue as the proportion of people living in cities and towns is projected to rise to 70% by 2050^1^ with the expansion of urban areas outpacing population growth^2^. Urban areas exist as a mosaic of buildings, grey infrastructure and greenspaces, with the area of sealed surface often exceeding 50% of a city or town’s extent^3^. The high proportion of impervious surface, with differing radiative, thermal, aerodynamic and moisture properties, results in elevated air temperatures compared to the surrounding rural landscape, commonly known as the urban heat island (UHI) effect^4^,^5^. The effects of the UHI are most apparent at night^6^ and during the summer months^7^; and vary between cities^5^, with temperature increases compared to the rural landscape sometimes reaching 6–12 °C^8^. Whilst the influence of the UHI on global temperatures is small compared to greenhouse gas induced climate change, it is not insignificant when combined with other changes in land-cover^6^.

Elevated temperatures can have significant public health effects particularly when extreme heat events result in the formation of stagnant warm air masses in urban areas^9^. In Europe in the summers of 2003 and 2013, extreme heat events directly resulted in serious health problems^10^,^11^, the 2003 the heatwave being directly responsible for 14,800 deaths in France, mostly in urban areas^10^. Such weather extremes, which are exacerbated in urban areas, will increase in frequency and severity due to climate change as exemplified in the UK by 70% of the warmest and 88% of the wettest seasonal records since 1910 being recorded since 2000^12^. In many cities and towns, globally, increases in urban temperatures have already exceeded projections for average temperature increases in response to climate change^13^. In addition to health effects these raised temperatures in urban areas can have consequences for energy use, for example, in the USA it is estimated that every 1 °C rise in temperature results in an increase in a city’s energy demand, for air conditioning, by 2–4%^4^.

Urban greenspaces can mitigate the effects of the UHI by changing the surface energy balance of the system^14^,^15^,^16^,^17^. Evapotranspiration from urban greenspaces can cool leaf surfaces and air temperature as the energy from solar radiation is stored as latent, rather than sensible heat^14^,^15^,^16^,^17^. Grass in an experimental plot was found to reduce maximum surface temperature by up to 24 °C when compared to concrete^16^. Similarly, lower soil temperatures at equivalent depths were observed beneath urban greenspace covered with grass when compared to a concrete covered surface in Nanjing, China^18^. Trees in an urban area may further mitigate elevated temperatures, particularly at the ground surface providing shade^14^,^16^,^17^, but they may also retain heat at night by restricting movement of warm air and reducing emissions of long wave radiation when compared to grassland^16^,^19^. However, urban greenspaces vary greatly in size and vegetation surface cover and exist as a mosaic within built infrastructure and thus their cooling effect is difficult to predict. For example, in Phoenix, USA, analysis of remotely sensed land surface temperature data revealed that clusters of grass and trees decreased land surface temperatures more than dispersed greenspaces^17^. In Taipei, Taiwan, parks greater than 3 ha in size were cooler than the surrounding area, whereas smaller parks had a much more variable effect^15^. Indeed, in some cases, where smaller parks were made of more than 50% paved surface they actually became warmer than the surrounding area at the time of temperature measurement^15^.

The effect of the UHI on ecosystem function in urban greenspaces is relatively poorly understood, but there is evidence that elevated air temperatures can alter plant and animal phenology. In Boston, USA, it was estimated that the growing season was extended by 20% compared to the surrounding rural environment^20^. The elevated air temperature may also affect the soil system, despite soil having a higher heat capacity than air^21^, with increases in soil temperature observed in cities in the USA^21^ and China^22^. Indeed, elevated temperatures have been observed in urban subsoils, to 20 m depth, but here the greatest increases were associated with buildings such as power stations^23^.

Despite a significant body of previous work on UHI, the effect of greenspace vegetation cover on soil surface temperature at a citywide scale, particularly the effect of trees and shrubs versus mown grassland have not been investigated. Greenspaces within urban areas comprise a mix of both domestically managed gardens and non-domestic greenspaces (such as large parks, patches of scrub, roadside verges) which differ both in patch-size and in the size trees and extent of their cover^24^ but the effect of these different types of greenspace land-use on soil temperatures are unknown. We were particularly interested in surface temperatures, not only because of their potential importance in heat exchange with the atmosphere, but also in influencing soil processes. Topsoil is the most biologically active part, soil organic matter is concentrated at the surface as is root biomass to intercept water and nutrient inputs. For example, 16 native and 5 amenity grass species in the USA showed highest rooting densities in the top 7.6 cm of soil profiles^25^. Furthermore, both diurnal and seasonal surface temperature changes are detectable down into the soil profile to at least 20–30 cm^26^,^27^, and surface measurements capture the extremes of these values.

Here, we provide a first systematic assessment of the interaction between vegetation type (herbaceous vegetation – predominantly lawns, versus trees and shrubs), land-use (domestic gardens versus non-domestic greenspace), and proximity to the urban core on soil surface temperature over 11 months, using data loggers distributed throughout our study city, Leicester UK (Fig. 1). Temperature was measured hourly in the top 1.5 cm of soil in order to understand the surface temperature fluctuations and extremes that urban soils experience. The daily “residual temperature” was calculated for each sensor from the differences between the mean daily temperature of the sensor and the mean of all the sensors on the same day, to determine effects of distance to the city centre, vegetation type, land-use and season on the soil surface temperatures.

We tested two hypotheses:

1. Vegetation cover affects soil surface temperature, specifically soils beneath trees and shrubs have lower summer temperatures than soils beneath herbaceous vegetation.
2. Proximity to city centre increases soil surface temperature especially in the summer.

## Results

Trees and shrubs in non-domestic greenspace had a substantial effect in reducing soil surface temperature variation compared herbaceous vegetation, whereas woody vegetation in domestic gardens only slightly reduced mean daily temperatures, and tended to increase temperature extremes in March and April compared to lawns and other herbaceous vegetation (Fig. 2). In the non-domestic greenspaces the effects of woody vegetation were most apparent during the summer months, when average temperature beneath herbaceous vegetation was more than 3 °C higher than beneath trees and shrubs; 17.2 and 14.1 °C respectively (Table 1). The largest effects were on summer mean maximum daily temperatures which ranged from 20.9 °C in the non-domestic herbaceous greenspace to 15.2 °C under trees and shrubs in the same land-use category, a decrease of 5.7 °C (Table 1). On many days maximum temperatures in grasslands exceeded 30 °C, whereas this temperature was never reached under woody vegetation in non-domestic greenspaces (Fig. 2, Table 1). In domestic gardens the overall effects of trees and shrubs decreased maximum temperatures on average by only 2.2 °C (Table 1).

Beneath herbaceous vegetation mean residual summer temperature was generally positive in the summer and negative in the winter, whereas beneath trees and shrubs mean residual temperature is generally negative in the summer and positive in the winter, this is most pronounced in the non-domestic land-cover classes (Fig. 3). The overall amplitude of mean residual temperature variation across the year was more than double in herbaceous vegetation than under woody vegetation indicating the effectiveness of trees and shrubs in buffering temperature extremes especially in the summer.

Evidence of an UHI is provided by the residual temperature at each location, averaged over 11 months, being highest in the city centre and declining with distance towards the edge of the city (fitted black lines in Fig. 4a; see Supplementary Table S1 for model rankings, top model sets and average coefficients). Differences in the slope of this decline between land-use and land cover types are negligible and equate to an UHI temperature increase of between 0.60–0.64 °C over 5 km from the suburbs to the city centre (Fig. 4a). Differences in the intercepts of the fitted lines reflect the effects of land-use and land cover on temperatures averaged over 11 months, and calculations of the overall effects of greenspace categories reveal modest overall annual cooling effects of woody vegetation versus herbaceous vegetation of 0.6 °C for domestic gardens and 0.8 °C for non-domestic greenspaces.

In contrast to the results for annual averages (Fig. 4a), models fitted to the data divided into winter and summer seasons (Fig. 4b) reveal pronounced differences between land-use types and seasons on the influence of distance from the city centre on residual soil surface temperatures. In winter, temperatures on domestic land decrease with distance from the city centre whilst temperatures on non-domestic land do not change with distance (blue lines in Fig. 4b). The reverse is true in the summer, when soil temperatures on non-domestic land show a strong decline of 1.4 °C under herbaceous vegetation and 1.5 °C under woody vegetation over the 5 km from city centre to outskirts of the city, whilst temperatures on domestic land tend to slightly increase away from the city centre (yellow lines in Fig. 4b). Importantly, the fitted models indicate for non-domestic greenspace a major cooling effect of trees that remains consistent with distance from the city centre and causes residual soil temperatures to be reduced by an average of 2.9 °C over the summer period (Fig. 4b).

## Discussion

**Figure 1:**
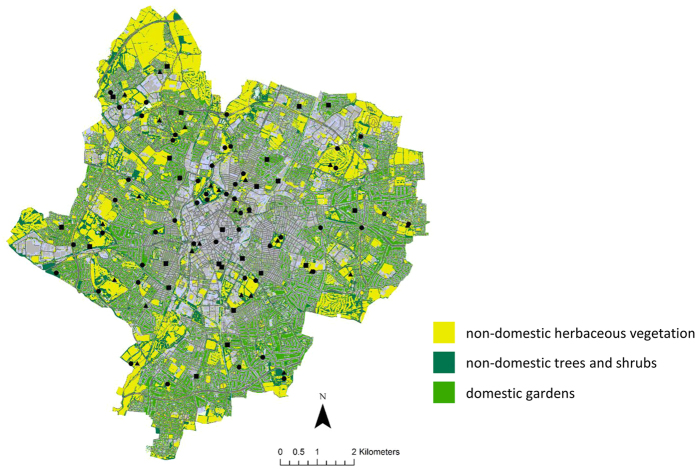
Map displaying greenspace coverage within the city of Leicester and the distribution of data loggers across the city. Closed squares indicate location of data loggers in domestic gardens, closed triangles indicate location of data loggers beneath non-domestic herbaceous vegetation, and closed circles indicate location of data loggers beneath non-domestic trees and shrubs. Map created in ArcGIS 10, using the LandBase dataset provided by Infoterra and MasterMap supplied by Ordnance Survey.

**Figure 2:**
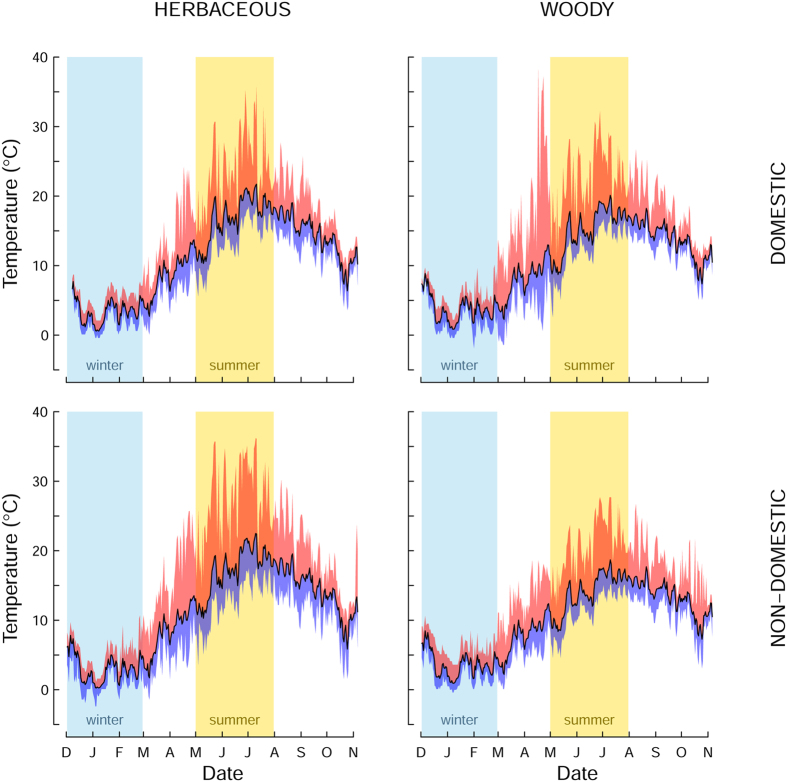
Average annual fluctuations in soil temperature for all data loggers beneath domestic herbaceous vegetation, domestic trees and shrubs, non-domestic herbaceous vegetation, non-domestic trees and shrubs. Black line represents mean daily temperature, bounded by red shading indicating maximum daily temperature and blue shading indicating minimum daily temperature.

This systematic study of soil surface temperature is the first at a citywide scale. It provides the first evidence of a decline in soil surface temperature with increased distance from a city centre observed throughout vegetation-class and land-use (Fig. 4a). This was as expected as the maximum effect of the UHI is generally in the dense city core^28^. Research has demonstrated that the larger the area of urban greenspace the greater the cooling effect^15^,^17^ and in general, in the city of Leicester the larger patches of greenspace are on the outskirts of the city in non-domestic land^29^. This suggests that with increased distance from the city centre, the increased proportional coverage of greenspace within the city (Fig. 1) dampens the UHI effect on soil surface temperature. Elevated urban soil temperatures in comparison to rural soils have been observed in both China^18^ and the USA^21^, and effects of urban greenspace on surface temperature have previously been modelled in Merseyside, UK giving a prediction of 7 °C higher temperatures in areas with only 15% greenspace coverage when compared to those with 50% coverage^7^.

**Figure 3:**
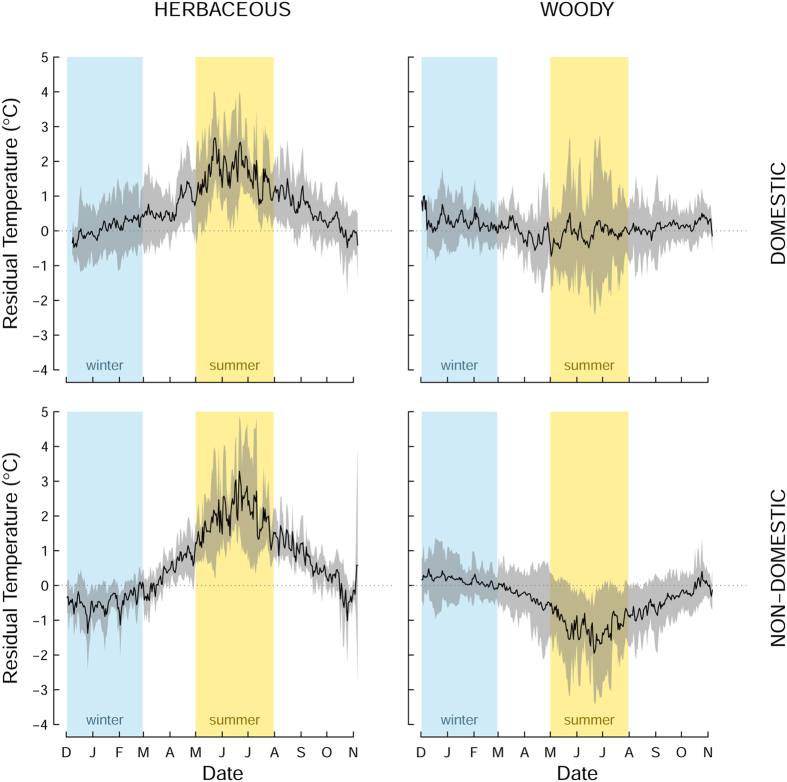
Average annual fluctuations in residual soil temperature (mean per-logger daily temperature minus mean daily logger temperature for all loggers) beneath domestic herbaceous vegetation, domestic trees and shrubs, non-domestic herbaceous vegetation, non-domestic trees and shrubs. Black line represents mean residual soil temperature bounded by grey shading indicating ±1 standard error. Vertical blue bands indicate winter months and vertical yellow bands indicate summer months.

**Figure 4:**
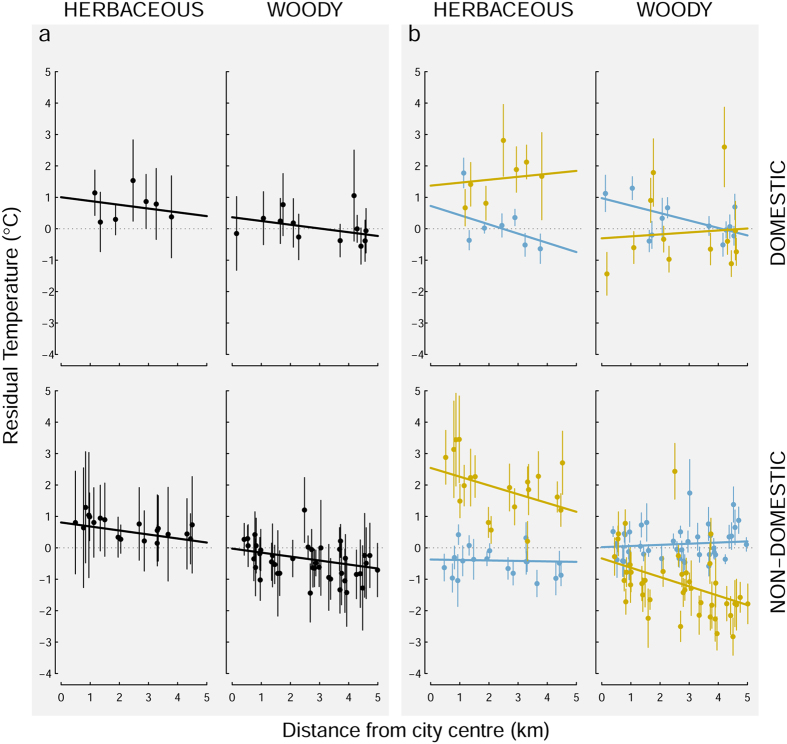
Effect of distance from city centre on (**a**) mean annual residual soil temperature (mean logger daily temperature minus mean daily logger temperature for whole logger dataset) – represented by the black data, and (**b**) mean summer residual soil temperature (May, June, July) – represented by the yellow data, and mean winter residual soil temperature (December, January, February) – represented by the blue data, beneath domestic herbaceous vegetation, domestic trees and shrubs, non-domestic herbaceous vegetation, non-domestic trees and shrubs. Error bars are ±1 standard error.

Within domestic land the mean residual soil temperature over the year fluctuated less and was closer to zero (average annual soil surface temperature for the whole dataset) than within non-domestic greenspace beneath both herbaceous and woody vegetation (Fig. 3). This could be attributed to the small patch size of urban gardens surrounded by buildings and linear features such as fences, hedges and walls, which, similar to the effect of trees, will increase shading and reduce surface heat loss at night through protection from the loss of long wave radiation^15^,^19^. In addition, woody vegetation in gardens had a much smaller effect in reducing soil surface temperatures in the summer. There are several likely reasons for this. Our previous studies of the composition of vegetation in the urban greenspaces of Leicester have shown that the density of trees in non-domestic greenspaces (average 986 ha^−1^) is almost 8 times higher than the 121 trees ha^−1^ in domestic gardens, and the mean size of each tree in gardens quantified by above-ground carbon storage is only 53 kg whereas in non-domestic greenspaces each tree holds an average of 98 kg of carbon^24^. As a consequence, the height and density of tree canopies in domestic gardens and the shading they produce will be generally less than for trees in non-domestic greenspaces, which includes stands of woodland.

The greatest UHI effect on soil surface temperature was observed during the summer months, which is consistent with research conducted in Nanjing, China^18^. The effects of woody vegetation in non-domestic green spaces supports previous findings that trees and shrubs are able to cool the urban environment and have potential to mitigate the UHI effect^4^,^21^, and provides evidence that this effect extends into the soil system. Much research has highlighted the cooling effect of greenspaces in urban ecosystems, however here we demonstrate in the soil system over nearly an entire year, the direct effect of trees and shrubs when compared to herbaceous vegetation (typically lawns) within the same greenspaces. The most striking effect of trees and shrubs was in non-domestic greenspace during the summer, where mean maximum soil temperature was 5.7 °C lower than beneath herbaceous vegetation. The timing of this effect coincides with the greatest potential risk associated with the UHI, heat related illness and death associated with extreme summer heat events^9^,^10^, and may be of increasing importance as the frequency of extreme weather events increases in response to climate change.

The effects of both vegetation cover and land-use on soil temperature further demonstrate the complexity of the urban ecosystem and the need for more detailed research at the extensive scale used herein to improve understanding of the interactions that drive soil carbon dynamics and nutrient cycling at a citywide scale. The small, relatively inexpensive data loggers we used recorded hourly soil temperatures over nearly one year demonstrating the potential of this new approach to combine high temporal and spatial sampling intensities to investigate the effect of the UHI on soil temperature in other urban areas, which is currently a neglected field of study. Previous research has typically focussed on a small number of sites studied over a detailed time-course; for example in Baltimore, USA, comparing four urban sites with two rural sites^21^, and in Nanjing, China, comparing a single urban site with a single rural one^18^. Conversely, in Nanjing, China, an extensive monitoring campaign of 600 sites, recorded soil temperature at one individual time-point, within a 3 day period^30^.

Our research further highlights the important role of urban trees in ecosystem service provision, as in addition to mitigating the UHI effect on soil surface temperature, they can also reduce soil bulk density which can help mitigate urban flooding^31^, increase carbon storage^24^,^32^, intercept pollution and improve air quality^33^. Furthermore, we demonstrate the importance of improving understanding of the effect of the UHI on the soil system, as elevated soil temperatures may increase rates of organic matter decomposition and alter nutrient cycling^34^,^35^. This is of particular concern as urban soils have recently been shown to contain strategically important stocks of carbon at a national scale^32^,^36^. Indeed, increases in soil respiration have been measured in a residential lawn when compared to an agricultural system in the USA^37^, and soil temperature explained 76% of the variation in soil respiration in a mixed urban forest in Beijing^38^. In addition, elevated soil temperatures may directly affect urban plant phenology having potential consequences for ecosystem service provision. This is of particular importance as global warming is likely to further exacerbate the UHI effect on urban soils.

## Methodology

### Study area

The study area for this research was Leicester, a mid-sized UK city in the East Midlands of England (52°32′N, 1°08W), with a human population of *c.* 330,000^39^. The East Midlands of the UK experiences a temperate climate, receiving 606 mm of precipitation annually and average annual daily minimum and maximum temperatures of 5.8 and 13.5 °C respectively^40^. Leicester covers an area of *c*. 73 km^2^ (as defined by the unitary authority boundary), of which 56% is greenspace and 44% covered by buildings or impervious surface.

### Soil temperature survey

Greenspace land-use in Leicester was determined in a GIS using the MasterMap dataset, supplied by Ordnance Survey and was split into either domestic gardens or non-domestic greenspace. Vegetation cover classes within the greenspace of Leicester were determined in GIS using the LandBase data supplied by Infoterra, there were two vegetation cover classes - herbaceous vegetation, shrubs and trees - in the two different land-uses types - non-domestic and domestic greenspace. A GIS was used to create five 1 km wide concentric zones, radiating out from the city centre. Within each zone data loggers were deployed at five randomly generated sites in the non-domestic herbaceous vegetation and trees and shrub category. For domestic gardens, a street layer was created in the GIS and used to select roads at random within each zone. Each of these roads was visited and, if there were residential properties present and authorization from a householder was granted, a data logger was deployed within the back garden of the household (see Fig. 1 for distribution of data loggers across the city). The data loggers, iButtons, recorded hourly soil temperature, for approximately 11 months with 0.5 °C accuracy. They were buried at approximately 1.5 cm soil depth, were deployed between November and December 2009 and collected in during December 2010 and January 2011. The shallow depth of burial minimised disturbance of greenspaces, made the approach suitable for securing agreements for deployment in private gardens, and facilitated the recovery of iButtons, 79/100 being relocated and having complete data.

### Statistical Analysis

Mean temperature per data logger per day was calculated from hourly temperature recordings, giving 79 separate 11-month temperature time series (one per data logger). These values were then used to calculate the daily “residual temperature”, by mean-centring according to overall daily mean temperature: each data point is the mean daily temperature for a specific data logger, minus the mean daily temperature across all loggers for that day. This measure accounts for the fact that absolute temperature varies according to other factors such as the time of year and the weather. Positive residual temperatures indicate recordings of higher value than the citywide mean for that day, whilst negative residual temperatures indicate recordings of lower value.

Three sets of data were analysed: the full data set, and a subset each for winter (days in December, January and February) and summer (days in May, June and July). Models fitted were Gaussian linear mixed effects (lme) models using maximum likelihood. Fixed effects were distance from city centre (continuous, in metres), land-use type (factorial, two levels: domestic or non-domestic), and vegetation type (factorial, two levels: herbaceous or woody i.e. shrubs and trees). Partial autocorrelation functions (pacfs) of the 79 individual time series indicated that residual temperature was non-independent up to a 3-day lag. A random intercept was fitted to data logger ID (factorial, seventy-nine levels) and the residuals were adjusted using an autoregressive lag-3 process to account for this non-independence. All possible combinations of fixed effects and their interactions were assessed: this includes the full model consisting of a three-way interaction between the fixed effects, and all possible subsets of this model (19 possible combinations per data set of fixed effect variables and their two-way interactions). Models were ranked according to their ΔAIC (Akaike information criteria), and averaged model coefficients were calculated using coefficients from models that ranked in the top 5% ΔAIC scores. All statistical analyses were conducted in R^41^: pacfs were calculated using the pacf function in the stats package; lme models were fitted using the lme function of the nlme package, and they were ranked, subsetted and averaged using the dredge, get.models and model.avg functions respectively, of the MuMIn package. Model rankings, top model sets, and average coefficients can be found in Supplementary Table S1.

## Additional Information

**How to cite this article**: Edmondson, J. L. *et al.* Soil surface temperatures reveal moderation of the urban heat island effect by trees and shrubs. *Sci. Rep.* **6**, 33708; doi: 10.1038/srep33708 (2016).

## Supplementary Material

Supplementary Information

## Figures and Tables

**Table 1:** Descriptive statistics for minimum, mean, and maximum daily temperatures during the summer (May, June, July) across land-use and vegetation cover classes.

		Domestic herbaceous	Domestic tree & shrub	Non-domestic herbaceous	Non-domestic tree & shrub
Minimum temperature	Mean (±1 S.E.)	14.6 (0.3)	13.7 (0.2)	14.4 (0.1)	13.0 (0.1)
	Minimum	5.6	5.1	6.2	4.6
	Maximum	20.1	20.7	21.1	21.2
	Range	14.5	15.6	14.9	16.6
Mean temperature	Mean (±1 S.E.)	16.6 (0.4)	15.2 (0.4)	17.2 (0.2)	14.1 (0.2)
	Minimum	9.0	7.5	9.4	7.1
	Maximum	24.2	24.7	26.9	24.2
	Range	15.2	17.2	17.5	17.1
Maximum temperature	Mean (±1 S.E.)	19.5 (0.8)	17.3 (0.7)	20.9 (0.5)	15.2 (0.2)
	Minimum	9.2	8.1	10.2	7.1
	Maximum	35.7	32.2	36.2	27.7
	Range	26.5	24.1	26.0	20.6
Sample size		8	11	19	41
