# Supplementary material for: Soil surface temperatures reveal moderation of the urban heat island effect by trees and shrubs
Source: Sci Rep. 2016 Sep 19;6:33708. doi: 10.1038/srep33708 (PMC5027384; doi:10.1038/srep33708)
Supplement: Supplementary Information [file srep33708-s1.pdf]

Supplementary Material

Soil surface temperatures reveal moderation of the urban heat island effect by trees and shrubs

J.L. Edmondson, I. Stott, Z.G. Davies, K.J. Gaston, J.R. Leake

Table S1: OVERALL mean daily temperature: AIC rankings from all models. A '+' indicates a variable or interaction that is included in a model. A '*' indicates a model that is included in the average model, i.e. where ΔAIC<(0.05*max(ΔAIC)). Models are listed in order of increasing AIC, therefore in order of decreasing likelihood.

Model terms								Model likelihoods				
Distance from city centre (km)	Vegetation (herbaceous or woody)	Land-use (domestic or non-domestic)	Distance: Vegetation interaction	Distance: Land-use interaction	Vegetation: Land-use interaction	Distance: Vegetation:Land-use interaction		df	log-likelihood	AIC	ΔAIC	weight
+	+	+			+		*	10	-9953.64	19927.30	0.00	0.279
+	+	+					*	9	-9955.11	19928.20	0.95	0.174
+	+	+		+	+		*	11	-9953.36	19928.70	1.44	0.136
+	+	+	+		+		*	11	-9953.56	19929.10	1.85	0.110
+	+	+		+			*	10	-9954.62	19929.20	1.96	0.105
+	+	+	+					10	-9955.01	19930.00	2.74	0.071
+	+	+	+	+	+			12	-9953.31	19930.60	3.35	0.052
+	+	+	+	+				11	-9954.56	19931.10	3.84	0.041
+	+	+	+	+	+	+		13	-9953.25	19932.50	5.24	0.020
+	+							8	-9959.36	19934.70	7.44	0.007
+	+		+					9	-9959.24	19936.50	9.20	0.003
	+	+			+			9	-9959.70	19937.40	10.13	0.002
	+	+						8	-9960.81	19937.60	10.35	0.002
	+							7	-9964.01	19942.00	14.75	0.000
+		+						8	-9976.36	19968.70	41.45	0.000
+		+		+				9	-9976.22	19970.40	43.17	0.000
+								7	-9979.58	19973.20	45.88	0.000
		+						7	-9981.61	19977.20	49.94	0.000
								6	-9984.05	19980.10	52.82	0.000

Coefficients of model average for daily mean temperature. Note that daily mean temperature is mean-centred and scaled individually for each day.

	Herbaceous Domestic	Woody Domestic	Herbaceous Non-domestic	Woody Non-domestic
Intercept	1.003315	0.36612	0.8077016	-0.0242679
Slope with dist	-0.1202223	-0.1192172	-0.1277303	-0.1267253

WINTER mean daily temperature (December, January and February): AIC rankings from all models. A '+' indicates a variable or interaction that is included in a model. A '*' indicates a model that is included in the average model, i.e. where ΔAIC<(0.05*max(ΔAIC)). Models are listed in order of increasing AIC, therefore in order of decreasing likelihood.

Model terms								Model likelihoods				
Distance (from city centre)	Vegetation (herbaceous or woody)	Land-use (domestic or non-domestic)	Distance: Vegetation interaction	Distance: Land-use interaction	Vegetation: Land-use interaction	Distance: Vegetation:Land-use interaction		df	log-likelihood	AIC	ΔAIC	weight
+	+	+	+	+			*	11	-666.80	1355.60	0.00	0.279
+	+	+	+	+	+		*	12	-665.91	1355.80	0.21	0.251
+	+	+		+			*	10	-668.45	1356.90	1.30	0.146
+	+	+		+	+		*	11	-667.65	1357.30	1.70	0.119
+	+	+	+	+	+	+	*	13	-665.68	1357.40	1.75	0.116
	+	+			+			9	-671.41	1360.80	5.21	0.021
	+	+						8	-672.76	1361.50	5.92	0.014
+	+	+	+		+			11	-669.90	1361.80	6.19	0.013
+	+	+			+			10	-671.02	1362.00	6.43	0.011
+	+	+						9	-672.34	1362.70	7.07	0.008
+	+	+	+					10	-671.36	1362.70	7.11	0.008
	+							7	-674.52	1363.00	7.45	0.007
+	+							8	-674.22	1364.40	8.84	0.003
+	+		+					9	-673.23	1364.50	8.85	0.003
+		+		+				9	-676.00	1370.00	14.39	0.000
		+						7	-679.00	1372.00	16.40	0.000
								6	-680.26	1372.50	16.91	0.000
+		+						8	-678.90	1373.80	18.19	0.000
+								7	-680.19	1374.40	18.78	0.000

Coefficients of model average for daily minimum temperature. Note that daily minimum temperature is mean-centred and scaled individually for each day.

	Herbaceous Domestic	Woody Domestic	Herbaceous Non-domestic	Woody Non-domestic
Intercept	0.7270059	0.9740042	-0.3717211	0.0206315
Slope with dist	-0.2939699	-0.2371177	-0.014946	0.03703782

SUMMER mean daily temperature (May, June and July): AIC rankings from all models. A '+' indicates a variable or interaction that is included in a model. A '*' indicates a model that is included in the average model, i.e. where ΔAIC<(0.05*max(ΔAIC)). Models are listed in order of increasing AIC, therefore in order of decreasing likelihood.

Model terms								Model likelihoods				
Distance (from city centre)	Vegetation (herbaceous or woody)	Land-use (domestic or non-domestic)	Distance: Vegetation interaction	Distance: Land-use interaction	Vegetation: Land-use interaction	Distance: Vegetation:Land-use interaction		df	log-likelihood	AIC	ΔAIC	weight
+	+	+		+	+		*	11	-4385.84	8793.70	0.00	0.479
+	+	+	+	+	+		*	12	-4385.58	8795.20	1.49	0.228
+	+	+	+	+	+	+	*	13	-4385.43	8796.90	3.18	0.098
+	+	+		+			*	10	-4388.81	8797.60	3.93	0.067
+	+	+			+		*	10	-4388.89	8797.80	4.11	0.062
+	+	+	+	+			*	11	-4388.61	8799.20	5.53	0.030
+	+	+	+		+		*	11	-4388.80	8799.60	5.93	0.025
	+	+			+		*	9	-4392.61	8803.20	9.54	0.004
+	+	+					*	9	-4392.65	8803.30	9.62	0.004
+	+	+	+					10	-4392.61	8805.20	11.53	0.002
+	+							8	-4395.14	8806.30	12.61	0.001
	+	+						8	-4395.84	8807.70	14.00	0.000
+	+		+					9	-4395.12	8808.20	14.55	0.000
	+							7	-4397.84	8809.70	16.00	0.000
+		+						8	-4430.48	8877.00	83.29	0.000
+		+		+				9	-4429.50	8877.00	83.33	0.000
+								7	-4432.05	8878.10	84.41	0.000
		+						7	-4433.28	8880.60	86.89	0.000
								6	-4434.53	8881.10	87.37	0.000

Coefficients of model average for dail maximumy temperature. Note that daily maximum temperature is mean-centred and scaled individually for each day.

	Herbaceous Domestic	Woody Domestic	Herbaceous Non-domestic	Woody Non-domestic
Intercept	1.37293	-0.304961	2.543842	-0.336347
Slope with dist	0.0937175	0.06259265	-0.279203	-0.2968509
